# Study on the correlation between IL-12p70, IL-17A and migraine in children

**DOI:** 10.3389/fneur.2024.1347387

**Published:** 2024-01-31

**Authors:** Fan Yang, Hai-zhen Liu, Jia-ai Liu, Yao-yao Chen, Su-zhen Sun

**Affiliations:** ^1^Department of Pediatrics, Hebei Medical University, Shijiazhuang, China; ^2^International Liaison Office, Hebei Children’s Hospital, Shijiazhuang, China; ^3^College of Life Sciences, Hebei Normal University, Shijiazhuang, China; ^4^Department of Pediatric Neurology, Hebei Children’s Hospital, Shijiazhuang, China

**Keywords:** migraine in children, serum cytokines, IL-12p70, IL-17A, TNF-α, IL-4

## Abstract

**Objective:**

To compare the serum levels of 12 cytokines in migraine group, encephalitis with headache symptoms group, pneumonia without headache symptoms group and migraine subgroups to explore the cytokines associated with migraine in children and their levels.

**Methods:**

A total of 44 children with migraine, 27 children in the encephalitis group with headache symptoms and 44 children in the pneumonia group without headache symptoms were selected from January 2022 to August 2023 in Hebei Children’s Hospital. They were all tested for serum cytokines by immunofluorescence assay. The migraine group was further divided into subgroups according to different age, gender, course of disease, and presence of coinfection. The differences of serum cytokine levels among the above groups were compared, and the correlation analysis was carried out.

**Results:**

Except IL-5, there were no significant differences in the expression levels of other 11 inflammatory cytokines between migraine subgroups. Compared with encephalitis with headache symptoms group and pneumonia without headache symptoms group the serum levels of IL-4, TNF-α, IL-17A, and IL-12p70 were higher in migraine group than in pneumonia group, and the levels of IL-12p70 were higher than those in encephalitis group (*p* < 0.05). An increase in serum IL-12p70 (*OR* = 1.267, 95%CI 1.054–1.523, *p* = 0.012) and IL-17A (*OR* = 1.066, 95%CI 1.016–1.119, *p* = 0.010) levels had a significant effect on migraine.

**Conclusion:**

Elevated serum levels of IL-12p70 and IL-17A may increase the risk of migraine in children, which has certain diagnostic and predictive value.

## 1 Introduction

Headache is one of the most common neurological symptoms in children, with migraine being the most prevalent primary headache (1). Migraine is a disorder of cerebral dysfunction (2) with a high incidence and an upward trend from year to year (3). However, currently, the complex pathophysiological mechanism of this disease has not been fully explained, and there are limitations in diagnosis and treatment (4). Previous studies have suggested that inflammatory reactions play a certain role in the occurrence and development of adult migraine (5) or animal models, which are most on the pro-infammatory, such as cytokines IL-1β, IL-6, TNF-α, and CGRP unfolded. With the advances in science and technology, cytokine detection has been widely applied in clinical diagnosis and treatment, however its application value in the diagnosis of migraine in children needs to be further explored. Based on the current clinical application of 12 types of cytokine detection methods including IL-12P70 and IL-17A, this study mainly explored the cytokines related to the presence of migraine in children and their levels.

## 2 Materials and methods

### 2.1 General information

This study retrospectively collected 44 children with migraine who were hospitalized in Hebei Children’s Hospital from January 2022 to August 2023, with an average age of (9.80 ± 2.29) years. A total of 27 hospitalized children with encephalitis and headache and 44 hospitalized children with pneumonia but without headache were selected as the control group. They were all tested for serum cytokine levels. The research plan was reviewed and approved by the Ethics Committee of Hebei Children’s Hospital (Ethics approval number 202332).

### 2.2 Inclusion and exclusion criteria

Inclusion criteria: (1) All the children were of Han nationality, aged 5–14 years; (2) The diagnosis of migraine was in accordance with the diagnostic criteria of the International Classification of Headache Disorders, 3rd edition (ICHD-3); (3) All the children with encephalitis had clinical manifestations of headache and cerebrospinal fluid white blood cell count ≥10 × 10^6^/L; (4) No headache was reported in children with pneumonia; and (5) The encephalitis group with headache symptoms and pneumonia group without headache symptoms had no previous diagnosis of migraine.

Exclusion criteria: (1) Patients who had received hormone, immunoglobulin, and hemofiltration treatment before specimen collection; (2) Patients with immune system deficiency, diseases of blood system, genetic metabolic diseases, autoimmune diseases, and other basic diseases; and (3) Patients with dysfunction of vital organs.

### 2.3 Methods

The migraine group was divided into 5–9 years old group (*n* = 21) and 10–14 years old group (*n* = 23) according to age. According to sex, they were divided into male group (*n* = 25) and female group (*n* = 19). According to the course of disease, they were divided into <1 month group (*n* = 24) and ≥ 1 month group (*n* = 20). The patients were divided into non-co-infection group (*n* = 19) and co-infection group (*n* = 25) according to presence of co-infection. Non-migraine group selection: A total of 27 children with headache and encephalitis met the inclusion and exclusion criteria; a total of 44 children with pneumonia but without headache were selected by random number method.

Clinical data were collected, including sex, age, course of disease, and serum cytokines.

The methods for detection of cytokines were as follows: 2 mL venous blood was collected on an empty stomach within 24 h in the morning, and the blood samples were collected with standard test tubes, followed by natural coagulation at room temperature or centrifugation at 2,000–4,000 rpm for 20 min. Subsequently, 0.5 mL of the separated serum was taken, and detected by flow cytometry using multiple microsphere flow immunofluorescence method within 4 h. If it cannot be detected within 4 h, place it at 2–8°C for no more than 24 h after the above treatment. Test equipment model: Challenbio MateCyte2L6c, and the kit used was 12 cytokines combined detection kit (Jiangxi Saiji Biotechnology Co., Ltd.), which was operated according to the product instructions.

### 2.4 Observation indicators

The levels of 12 kinds of serum cytokines were compared in migraine group, encephalitis group with headache symptoms and pneumonia group without headache symptoms, and also in migraine subgroups.

### 2.5 Statistical methods

SPSS26.0 was used for statistical analysis, and GraphPad Prism10 statistical plotting software was used for analysis and plotting. The measurement data of normal distribution are described as mean ± standard deviation [( $\bar{X}\pm$ SD)]. The difference between the two groups was evaluated by independent sample *t* test. The median (interquartile distance) [M (IQR)] description was used when the distribution did not conform to the normal distribution. The Mann–Whitney test was performed to evaluate the difference between the two groups, and the Kruskal-Wallis test was used to evaluate the difference between groups. *p* < 0.05 was considered statistically significant. The relevant cytokines were included in the binary multivariate Logistic regression equation, and the prediction model was constructed. The prediction probability of migraine was calculated, and the ROC curve was drawn to analyze its diagnostic value for hemicephalic pain.

## 3 Results

### 3.1 Comparison of serum cytokine levels in children with migraine with respect to different ages, different genders, different courses of disease, and presence or absence of co-infection

The difference in IL-5 in different age groups was statistically significant (*p* < 0.05), which was higher in the low age group than in the high age group, whereas the other indicators showed no significant difference, as shown in Tables 1 2.

**Table 1:** Comparison of cytokine levels in children with migraine in different age groups and different sex groups.

Cytokines (pg/mL) M(IQR)	Migraine group (*n* = 44)	Classification of age		Classification of age gender	
		5–9 years (*n* = 21)	10–14 years (*n* = 23)	*M* (*n* = 25)	*F* (*n* = 19)
IL-2	2.16 (1.62)	2.20 (1.60)	1.99 (1.96)	2.01 (1.98)	2.17 (1.44)
IL-4	3.52 (3.97)	3.39 (3.85)	4.09 (4.35)	3.62 (5.06)	3.38 (3.69)
IL-6	6.08 (4.63)	6.45 (6.09)	5.55 (2.94)	6.08 (6.44)	5.92 (2.89)
IL-10	4.42 (3.00)	4.49 (2.90)	4.37 (3.35)	4.41 (3.58)	4.88 (2.19)
TNF-α	5.60 (4.23)	5.41 (4.09)	5.67 (6.28)	5.45 (6.60)	5.93 (3.12)
IFN-γ	7.46 (4.51)	7.47 (5.24)	7.41 (5.73)	7.41 (5.51)	7.99 (3.67)
IL-17A	16.40 (16.16)	15.88 (10.09)	18.57 (23.06)	17.97 (21.90)	15.75 (11.36)
IL-1β	2.58 (3.41)	3.49 (3.28)	2.47 (3.70)	3.05 (5.05)	2.47 (3.15)
IL-5	1.04 (1.63)	1.55 (1.54)	0.93 (0.86)^#^	1.00 (0.90)	1.55 (1.65)
IL-12p70	5.10 (2.91)	4.92 (3.80)	5.10 (3.00)	4.90 (4.08)	5.29 (3.00)
IFN-α	3.92 (3.95)	5.91 (5.53)	3.40 (4.08)	3.81 (3.87)	4.68 (4.12)
IL-8	2.95 (5.03)	3.35 (10.00)	2.93 (3.89)	2.93 (4.68)	3.35 (6.46)

^#^means *p* < 0.05 for comparison between the two groups of migraine children grouped by age.

**Table 2:** Comparison of cytokine levels in children with migraine in different disease course groups and whether they were combined with infection.

Cytokines (pg/mL) M(IQR)	Migraine group (*n* = 44)	Grouping by course of disease		Grouping by co-infection or not	
		<1 month (*n* = 24)	>1 month (*n* = 20)	Unco-infection (*n* = 19)	Co-infection (*n* = 25)
IL-2	2.16 (1.62)	2.28 (2.22)	2.03 (1.43)	2.39 (2.07)	2.05 (1.32)
IL-4	3.52 (3.97)	3.86 (4.67)	3.43 (3.01)	4.34 (4.13)	3.38 (3.67)
IL-6	6.08 (4.63)	5.74 (4.16)	6.27 (6.95)	6.45 (5.20)	6.07 (3.64)
IL-10	4.42 (3.00)	4.15 (2.70)	4.71 (3.26)	4.96 (2.84)	4.06 (2.58)
TNF-α	5.60 (4.23)	6.05 (6.35)	5.47 (3.15)	6.35 (5.65)	5.41 (4.35)
IFN-γ	7.46 (4.51)	6.92 (4.55)	7.49 (4.36)	7.99 (4.60)	7.18 (5.68)
IL-17A	16.40 (16.16)	15.82 (7.80)	18.11 (26.71)	16.39 (15.54)	16.41 (19.48)
IL-1β	2.58 (3.41)	2.76 (4.37)	2.47 (3.31)	2.87 (2.92)	2.47 (4.25)
IL-5	1.04 (1.63)	1.07 (0.87)	1.04 (2.19)	1.12 (1.69)	1.02 (1.59)
IL-12p70	5.10 (2.91)	5.19 (4.37)	5.06 (2.24)	5.78 (4.24)	4.70 (3.29)
IFN-α	3.92 (3.95)	3.74 (3.96)	4.76 (5.01)	4.68 (4.12)	3.44 (3.85)
IL-8	2.95 (5.03)	3.84 (5.47)	2.31 (4.65)	3.43 (5.76)	2.85 (5.02)

### 3.2 Comparison of 12 kinds of serum cytokines between migraine group, encephalitis group with headache symptoms, and pneumonia group without headache symptoms

**Figure 1:**
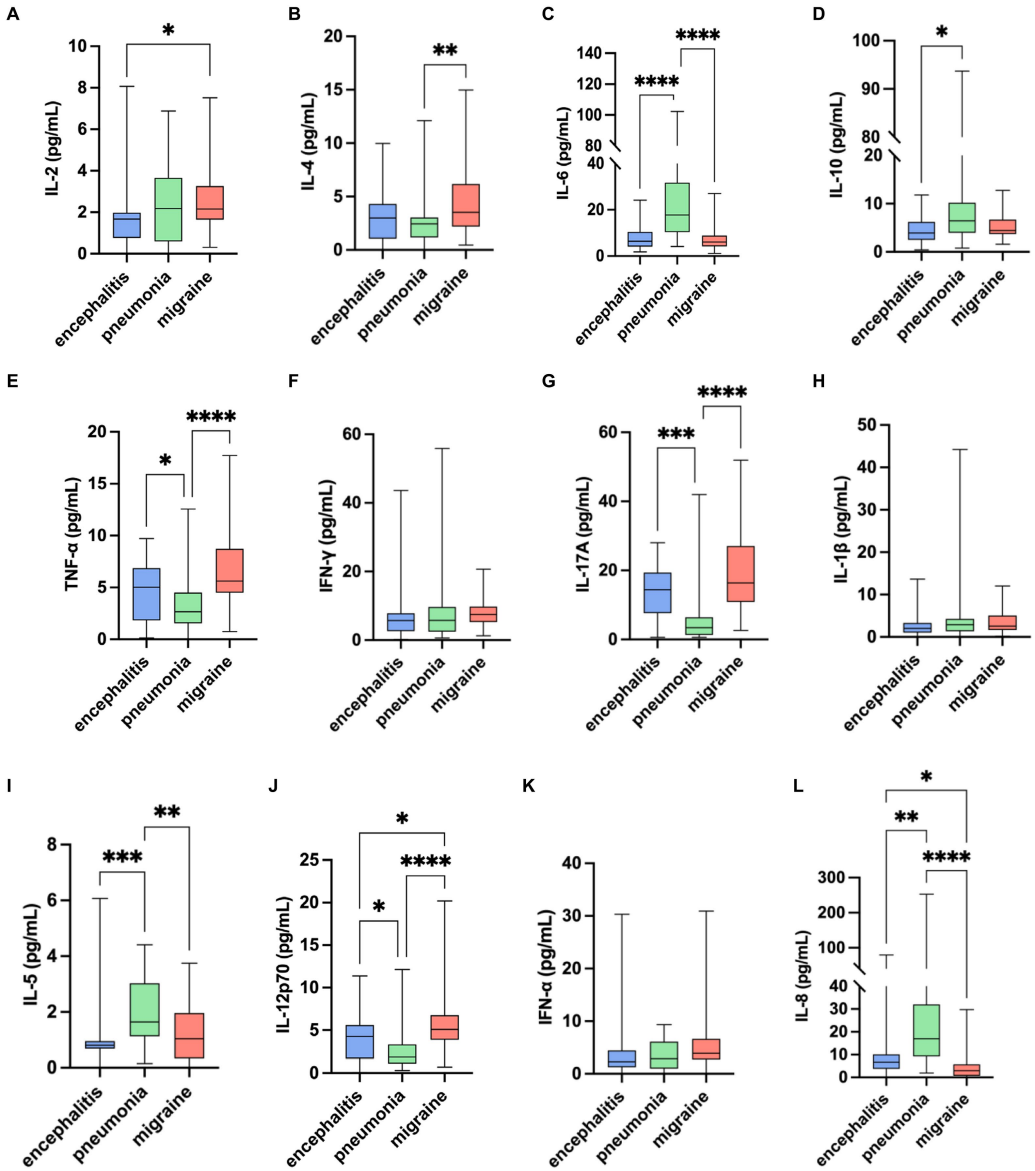
Comparison of 12 serum cytokines levels among migraine, encephalitis with headache symptoms, and pneumonia without headache symptoms. ^*^p < 0.05, ^**^p < 0.01, ^***^p < 0.001, ^****^p < 0.0001: pairwise comparison between groups.

Based on IL-2 in migraine group [2.16 (1.62) pg/mL], in encephalitis with headache group [1.67 (1.22) pg/mL], and in pneumonia without headache group [2.18 (3.07) pg/mL], there were statistical differences in the overall distribution of IL-2 among the three groups (*H* = 6.80, *p* = 0.033). There was significant difference between the moderate headache group and the encephalitis with headache group (*p* = 0.027), and there was no significant difference between the migraine group and the pneumonia without headache group, and between the encephalitis with headache group and the pneumonia without headache group (*p* > 0.05) (Table 3; Figure 1).

**Table 3:** Comparison of 12 serum cytokines levels between migraine and encephalitis with headache symptoms and pneumonia without headache symptoms.

Cytokines (pg/mL) M(IQR)	Migraine group (*n* = 44)	Encephalitis group (*n* = 27)	Pneumonia group (*n* = 44)	Kruskal-Wallis T	
				*H*	*p*
IL-2	2.16 (1.62)	1.67 (1.22)	2.18 (3.07)	6.80	0.033
IL-4	3.52 (3.97)	2.99 (3.25)	2.44 (1.87)	12.81	0.002
IL-6	6.08 (4.63)	6.36 (6.27)	17.75 (21.33)	39.17	0.000
IL-10	4.42 (3.00)	3.91 (3.72)	6.43 (6.23)	8.62	0.013
TNF-α	5.60 (4.23)	5.02 (5.03)	2.66 (2.97)	28.08	0.000
IFN-γ	7.46 (4.51)	5.70 (5.22)	5.79 (7.21)	5.82	0.054
IL-17A	16.40 (16.16)	14.45 (11.76)	3.43 (5.10)	39.40	0.000
IL-1β	2.58 (3.41)	2.04 (2.32)	2.92 (2.92)	3.67	0.160
IL-5	1.04 (1.63)	0.81 (0.27)	1.64 (1.90)	17.72	0.000
IL-12p70	5.10 (2.91)	4.25 (3.93)	1.85 (2.22)	33.67	0.000
IFN-α	3.92 (3.95)	2.27 (3.20)	2.88 (5.12)	6.73	0.035
IL-8	2.95 (5.03)	6.68 (6.38)	16.99 (22.73)	49.62	0.000

As for IL-4 in migraine group [3.52 (3.97) pg/mL], in encephalitis with headache group [2.99 (3.25) pg/mL], in pneumonia without headache group [2.44 (1.87) pg/mL], there were statistical differences in the overall distribution of IL-4 levels among the three groups (*H* = 12.81, *p* = 0.002). There was significant difference between migraine group and pneumonia without headache group (*p* = 0.001), while there was no significant difference between migraine group and encephalitis with headache group, and between encephalitis with headache group and pneumonia without headache group (*p* > 0.05).

TNF-α in migraine group [5.60 (4.23) pg/mL], in encephalitis with headache group [5.02 (5.03) pg/mL], and in pneumonia without headache group [2.66 (2.97) pg/mL] showed that there were significant differences in the overall distribution of TNF-α levels among the three groups (*H* = 28.08, *p* < 0.0001). There were significant differences between the migraine group and the pneumonia without headache group (*p* < 0.0001), and between the encephalitis with headache group and the pneumonia without headache group (*p* = 0.037), whereas there was no significant significance between the migraine group and the encephalitis with headache group (*p* > 0.05).

IL-17A in migraine group [16.40 (16.16) pg/mL], in encephalitis with headache group [14.45 (11.76) pg/mL], and in pneumonia without headache group [3.45 (5.10) pg/mL] revealed that there were statistical differences in the overall distribution of IL-17A among the three groups (*H* = 39.40, *p* < 0.0001). There was significant difference between the migraine group and the pneumonia without headache group (*p* < 0.0001), and between the encephalitis with headache group and the pneumonia without headache group (*p* = 0.0003), whereas no significant difference was found between the migraine group and the encephalitis with headache group (*p* > 0.05).

It was found that IL-12p70 was [5.10 (2.91) pg/mL] in migraine group, [4.25 (3.93) pg/mL] in encephalitis with headache group, and [1.85 (2.22) pg/mL] in pneumonia without headache group, suggesting significant difference in the overall distribution of IL-12p70 among the three groups (*H* = 33.67, *p* < 0.0001). There was statistical difference between the migraine group and the pneumonia without headache group (*p* < 0.0001), and between the migraine group and the encephalitis with headache group (*p* = 0.045). Significant difference was noted between the encephalitis with headache group and the pneumonia without headache group (*p* = 0.026).

IL-6 and IL-5 in the migraine group were statistically different from those in the pneumonia group without headache. IL-8 in the migraine group was statistically different from those in the two control groups. But the levels of IL-6, IL-5, and IL-8 in the migraine group were lower than those in the control group. IL-10 between migraine group and control group showed there were no significant differences.

Despite significant difference in the overall distribution of IFN-α levels (*H* = 6.73, *p* = 0.035), there was no significant difference between groups (*p* > 0.05). There was no statistical difference in the overall distribution of IFN-γ and IL-1β levels among the three groups (*H* = 5.82, *p* = 0.054; *H* = 3.67, *p* = 0.160).

Kruskal-Wallis test among the three groups showed that the levels of IL-4, TNF-α, IL-17A, and IL-12p70 in children with migraine were higher than those in pneumonia without headache group, and the levels of IL-12p70 were also higher than those in children with encephalitis with headache and pneumonia without headache group.

### 3.3 Value of serum IL-4, TNF-α, IL-17A, and IL-12p70 in the diagnosis of migraine

**Figure 2:**
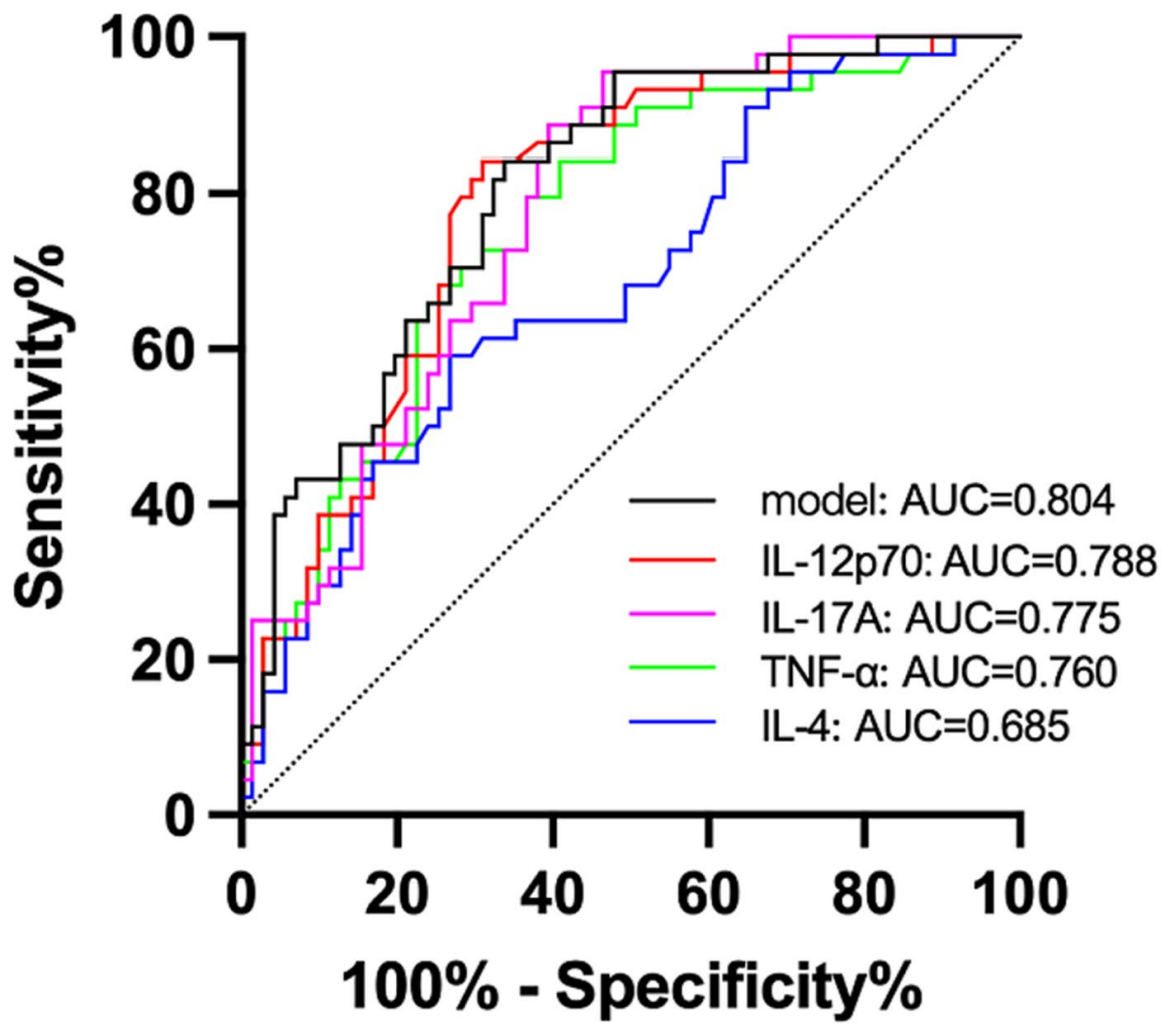
Value analysis of serum IL-4, TNF-α, IL-17A, and IL-12p70 in the diagnosis of migraine.

According to ROC analysis, IL-12p70 alone had the optimal predictive effect, while IL-4 had the worst predictive effect (Figure 2). Serum IL-12p70 was higher than 3.57 pg/mL (reference value 0–3.40 pg/mL), and the sensitivity and specificity in the diagnosis of migraine were 84.1 and 69.0%, respectively. When serum IL-17A level was >7.99 pg/mL (0–20.60 pg/mL), the sensitivity and specificity in migraine diagnosis were 88.6 and 60.6% respectively, and when serum IL-17A level was >20.60 pg/mL, the sensitivity and specificity were only 25.35 and 93.18%, respectively. As for serum TNF-α level > 3.97 pg/mL (0–4.60 pg/mL), the sensitivity and specificity in migraine diagnosis were 84.1 and 59.2% respectively; when serum IL-4 level was >4.60 pg/mL, sensitivity and specificity were 76.06 and 72.73%, respectively; when serum IL-4 level was >3.36 pg/mL (0–3.00 pg/mL), the sensitivity and specificity in migraine diagnosis were 59.1 and 73.2%, respectively.

IL-4, TNF-α, IL-17A, and IL-12p70 were included in the Logistic regression equation, and IL-17A and IL-12p70 were included in the final model; however, IL-4 and TNF-α had no predictive significance. The results suggested that an increase in serum IL-17A level had a significant effect on migraine (*OR* = 1.066, 95%CI 1.016–1.119, *p* = 0.010). The effect of increased serum IL-12p70 levels on migraine was statistically significant (*OR* = 1.267, 95%CI 1.054–1.523, *p* = 0.012). The ROC curve of the prediction model is shown in Figure 2 (black). The performance of the model was improved, and the AUC was 0.804 (0.725, 0.883). When the prediction probability of the prediction model was greater than 0.285, the Yoden index of migraine was evaluated to be the largest, and the sensitivity and specificity were 0.841 and 0.662, respectively.

When the ROC curve was drawn with headache as the state variable, the results showed that IL-17A alone had the optimal prediction effect, but the observational data did not fit well with the regression model.

## 4 Discussion

Cytokines are soluble signaling peptides secreted mainly by immune cells, which are key mediators of immune response and inflammation. Its role in the pathogenesis of migraine has been embraced by researchers (5). A meta-analysis showed that serum CRP, IL-1β, IL-6, and TNF-α were higher in migraine patients than in healthy controls, while serum IL-2 and IL-10 showed no significant differences (6). Li Jinhong found that the levels of serum INF-α, IL-2, IL-17, IL-12P70, TNF-α, IL-5, IL-1β, and IL-4 were higher in adult migraine patients than in normal control group, which changed with different stages of the disease. Specifically, IL-17 and TNF-α were higher in the medium-frequency group than in the low-frequency group, and had a downward trend in the chronic group. The levels of IL-2 and IL-12P70 decreased in chronic group than in the medium-frequency group (7).

In this study, there were 12 serum cytokines involved, namely IL-2, IL-4, IL-6, IL-10, TNF-α, IFN-γ, IL-17A, IL-1β, IL-5, IL-12P70, INF-α, and IL-8. The key findings were as follows: the serum levels of IL-4, TNF-α, IL-17A, and IL-12p70 were higher than those in the pneumonia without headache group, and the serum level of IL-12p70 was higher than that in the encephalitis with headache group. The prediction model suggested that the increase in serum IL-17A and IL-12p70 levels had a certain predictive value for the diagnosis of migraine, which was not completely consistent with reports in previous studies. This may be related to the fact that there was the small pediatric sample size in our study and that children with encephalitis and pneumonia, other than normal healthy children, were enrolled in the control group. The discovery of IL-6 and IL-5 might be interfered by the severity of different microbial infections and diseases. Although grouped comparison of presence of co-infection in patients with migraine was performed in this study, no significant cytokines were found. This suggested that infection did not affect overall cytokine level in the migraine group, which might be related to mild infections, but severe infections accounted for a larger proportion in the control group, especially in the pneumonia group.

IL-4 is an anti-inflammatory cytokine with neuroprotective, nutritive and immunosuppressive effects (8), which can induce M2 macrophages to produce opioid peptides. Through activating peripheral opioid receptors, mechanical hypersensitivity caused by neuropathy and pain will be relieved (9). This study found that serum IL-4 in the migraine group was higher than that in the pneumonia without headache group, but was not significantly different, as compared with the encephalitis group. This supported the need for IL-4 protection in both migraine and encephalitis; however, because there was no significant difference between the encephalitis group and the pneumonia group, the evidence strength was weak. TNF, as a key mediator of secondary central nervous system injury under acute injury and chronic inflammatory conditions, has been shown in animal experiments to cross the blood–brain barrier (BBB) into the brain via a specific transport system. In addition, under the stimulation of inflammation, microglia, astrocytes, and choroidal plexus ependymal cells can produce a large amount of TNF (10). In addition to powerful pro-inflammatory effect, TNF also mediates the basic protective function of the central nervous system (10). The results suggested that TNF-α exerted a harmful effect on the onset of headache in children, especially the attack of migraine, which was consistent with previous studies. Unfortunately, no significant predictive value for diagnosis was found. However, Rubino et al. firstly used pharmacogenetic methods to confirm that TNF-α polymorphisms in gene function significantly modulated the clinical response to NSAID administration during acute migraine attacks, further supporting the role of the pro-inflammatory cytokine TNF-α in the pathophysiological mechanism of migraine attack (11).

IL-17 is a cytokine expressed by T cells and eosinophils and is mainly activated by this cell membrane memory phenotype. IL-17A is a family member of IL-17, which is a signature cytokine in a set of CD4^+^ Th17 cells, has a protective effect on skin mucosal barrier and can defense against extracellular fungi and bacteria, as well as helping repair damaged tissues, showing the closest relationship with human health and disease (12). Impact of IL-17A on the nervous system is still in the preliminary stage of exploration. The main findings are that changes in cytokine regulatory environment may lead to the increase of T cells, which produces IL-17 under intestinal ecological imbalance conditions and exacerbates autoimmune diseases targeting the central nervous system (12). This study suggested that serum IL-17 concentration in children with migraine and headache or those with encephalitis and headache was significantly higher than that in children with pneumonia without headache, but there was no statistical difference between children with migraine and those with encephalitis.

IL-12p70, composed of p35 and p40 subunits, is a proinflammatory cytokine produced primarily by antigen-presenting cells and mediates type 1 helper T cell (Th1) differentiation. In neurobehavioral aspect, IL-12p70 may further mediate mechanical hyperalgesia in rodents through the action of endothelin and its B-type receptor (13). In another preclinical study, the analgesic effect of IL-12p40 was found but it was unclear whether IL-12p70 could cause pain (14). However, Oliveira et al. (15) found that serum level of IL-12p70 was higher in female migraine patients than in healthy control group, and that appropriate aerobic exercise could shorten the duration of migraine headache as well as lowering the serum level of IL-12p70 (16). In the early stage of neurodegeneration driven by astrocytes and macrophages/microglia, the inflammatory response of CD4^+^Th17 cells, CD4^+^Th1 cells, IFN-γ, IL-12, TNF-α, and NO led to axon injury and demyelination (17). In addition, magnetic resonance examination of the skull in clinical studies showed that several migraine sufferers had leukoencephalopathy, which shared the neuroinflammatory mechanism with neurodegenerative diseases such as multiple sclerosis (18). While, serum IL-12p70 concentration was significantly different between children with pediatric migraine and those with encephalitis and headache, suggesting that IL-12P70 played a more important role in the mechanism related to neuroinflammation of migraine. In a preclinical study, Chen et al. (19) further proved that nitroglycerin (NTG) induced peripheral IL-17A infiltration into medulla oblongata (MO) to activate microglia and induced calcitonin gene-related peptide (CGRP)-related neuroinflammation by increasing BBB permeability. Eventually, mechanical hyperalgesia and migraine attacks were induced by activation of the trigeminal caudate nucleus (TNC) (19). Whether the effect of IL-12p70 on migraine is mediated by microglia, peripheral immune cells, or both needs to be further investigated.

Several scholars currently consider that the evidence is insufficient to support the relationship between acute migraine attack and neuroinflammatory responses. Neurogenic neuroinflammation caused by the continuous release of neurotransmitters may be the core of chronic migraine (20). Although the serum samples in this study were collected during the onset of headache, they could only provide indirect evidence of effects. We conducted a comparison between different courses of migraine, but due to the sample size, 1-month medical history was set as the time node, and no statistical differences were found. Therefore, further research is needed to investigate the differences in cytokine levels between paroxysmal and chronic migraines, especially in intracranial levels. In the meantime, study results also suggested that serum IL-5 concentration in children with migraine was higher in the low age group than in the high age group, while no statistically significant differences in other cytokine levels were found in different subgroups of migraine.

In conclusion, serum levels of IL-4, TNF-α, IL-17A, and IL-12p70 are increased in children with migraine. Moreover, elevated serum IL-12p70 and IL-17A levels will also increase the risk of migraine in children, which has certain predictive value for migraine diagnosis.

## Data availability statement

The raw data supporting the conclusions of this article will be made available by the authors, without undue reservation.

## Ethics statement

The studies involving humans were approved by Medical Research Ethics Committee of Hebei Children’s Hospital. The studies were conducted in accordance with the local legislation and institutional requirements. Written informed consent for participation in this study was provided by the participants’ legal guardians/next of kin.

## Author contributions

FY: Data curation, Formal Analysis Funding acquisition, Methodology, Project administration, Writing – original draft, Writing – review & editing. H-zL: Formal Analysis, Writing – original draft. J-aL: Formal Analysis, Validation, Writing – review & editing. Y-yC: Formal Analysis, Validation, Writing – review & editing. S-zS: Conceptualization, Supervision, Writing – review & editing.
